# Disentangling the dynamics of social assistance: A linked survey—Register data cohort study of long-term social assistance recipients in Norway

**DOI:** 10.1371/journal.pone.0230891

**Published:** 2020-03-27

**Authors:** Kristian Heggebø, Espen Dahl, Kjetil A. van der Wel

**Affiliations:** 1 NOVA, OsloMet–Oslo Metropolitan University, Oslo, Norway; 2 Department of Social Work, Child Welfare, and Social Policy, Oslo Metropolitan University, Oslo, Norway; University of Jyvaskyla, FINLAND

## Abstract

Social assistance is a means-tested benefit that is supposed to be a short-term, temporary economic support. Understanding why some individuals are in repeated or continuous need of social assistance is thus of obvious policy relevance, but the dynamics of social assistance receipt remain poorly understood. In 2005, a survey among long-term recipients of social assistance in Norway collected data on (a) childhood disadvantages, (b) health status, (c) health behaviors, (d) psychological resources, and (e) social ties, in addition to basic sociodemographic information. This rich survey data has been linked with tax register data from 2005–2013, enabling us to explore the detailed characteristics of long-term social assistance recipients who are unable to reach financial self-sufficiency. Results from linear probability models show that surprisingly few of the 28 explanatory variables are statistically associated with social assistance dynamics, with two important exceptions: People with drug problems and immigrants both have a much higher probability of social assistance receipt. Yet overall, it is challenging to ‘predict’ social assistance dynamics, indicating that randomness most likely plays a non-negligible role. The 28 explanatory variables do a far better job in predicting both labor market success (employment), labor market preparation (work assessment allowance), and labor market withdrawal (disability benefit utilization). Thus, there seems to be something distinctive about the processes leading to continued social assistance recipiency, where randomness could be a more influential force.

## Introduction

Social assistance is the final safety net in the Norwegian welfare state, which is granted only if all other potential income sources are exhausted. It is an explicit aim that this means-tested benefit is a temporary economic support that promotes self-sufficiency [1]. Thus, social assistance is designed to be, and normally is, a transitory and short-term (e.g., 2–3 months) income source available for people who are in (temporary) need of help. Yet, a non-negligible minority receives social assistance for a prolonged period [2], and this group of *long-term social assistance recipients* is the focus of this study.

Previous research has established that individuals who received social assistance (SA) in the past have a heightened probability of doing so in the future as well [3, 4], and repeated spells are a common empirical finding [5, 6]. Cross-national differences are also evident, where e.g., Königs [7], using register data for 2001–2008 from Luxembourg, the Netherlands, Norway and Sweden, highlights that long-term SA receipt is a comparatively rare event in the two Nordic countries. This is also evident from official Norwegian statistics: In 2013, the last observational year of the current study, merely 11.38 percent of all recipients (N = 13 743/120 775) received social assistance for 12 months straight [2]. Thus, very long-term receipt of social assistance (e.g., consecutively for more than a year) is infrequent in Norway. Accordingly, people who have social assistance as their main income source for more than six months during a calendar year is considered as ‘long-term’ recipients in this paper.

The current study adds to the existing literature in two ways. First, by combining rich survey data with longitudinal follow-up in administrative registers, our study investigates how a number of individual factors–most of them unobserved in previous research–are statistically associated with social assistance dynamics. Second, in contrast to the narrow focus on SA entry/exit in the existing literature, we compare our empirical findings for social assistance recipiency with: (i) employment (i.e., labor market *success*), (ii) work assessment allowance (i.e., labor market *preparation*), and disability benefit utilization (i.e., permanent labor market *withdrawal*). Exit from SA receipt does not necessarily equal self-sufficiency in comprehensive welfare states such as Norway, where several income maintenance schemes are available. Furthermore, comparing results across alternative income sources will help determine whether different benefit- and employment ‘routes’ exists for long-term social assistance recipients according to their individual characteristics.

The 2005 survey data makes it possible to examine the importance of 28 explanatory variables on (a) childhood disadvantages, (b) health status, (c) health behaviors, (d) psychological resources, and (e) social ties, while controlling for basic sociodemographic covariates. Linking these survey data with the register data for 2005–2013, we have access to high-quality longitudinal information on the benefit careers and employment histories for a cohort of long-term social assistance recipients. We ask the following overarching research question: *To what extent are the 28 explanatory variables able to ‘predict’ the dynamics of social assistance for a cohort of long-term social assistance recipients in Norway*?

## Previous research

A large number of studies on social assistance dynamics has been conducted in several OECD countries during the past decades (see e.g., [7, 8, 9, 10]). However, due to the rather unique feature of Norwegian (and Nordic) patterns of social assistance receipt, we will only cover previous research from Nordic countries in the following. Numerous papers have investigated social assistance dynamics in Nordic welfare states. Dahl & Lorentzen [5] look at exit from social assistance in Norway, and find that those with lower education and immigration background have lower exit rates. Hansen [6] shows that 50 percent of natives end their social assistance reliance during the first year in Norway, as compared to about 35 percent of immigrants. Backman and Bergmark [11] find that labor market experience and previous SA spells are associated with exit from social assistance in Sweden. Another study from Sweden shows that those who receive SA for longer periods have low education, a history of lower incomes, receive higher amounts of SA, and are likely to have more children and to be single mothers [3].

Hyggen [12] use a linked Norwegian survey—register data material to investigate social assistance likelihood in youth and young adulthood. Parental resources played a key role for those aged 18–28 year: First, the higher the father’s educational level, the lower social assistance probability, and second, youths with both parents on social welfare benefits had a higher chance of social assistance receipt. In young adulthood (25–36 years), single parents were at a greater risk of social assistance receipt, and unemployment spells was also a significant predictor. Finally, those who had received social assistance in the past had a heightened probability of doing so in young adulthood as well. Lorentzen, Dahl & Harsløf [13] utilize Norwegian register data 1993–2004 to examine the impact of life events and social background characteristics. Marital breakdown, becoming a single provider and leaving the parental home are associated with higher social assistance likelihood. As to social background characteristics, (higher) parental education and family income were both linked with (lower) social assistance probability. In Finland, Ilmakunnas & Moisio [14] study determinants of social assistance among young adults (19–25 years) using register data for 2000–2012. Lack of educational qualifications, parental social assistance receipt, and moving out of the parental home at a young age are associated with risk of social assistance.

Turning to cross-national comparative studies, Wiborg and Møberg [15] analyze Danish and Norwegian register data for 1992–2003, and shows that the impact of parental education on social assistance likelihood among young adults is more pronounced in Denmark than in Norway. However, the association between own educational level and social assistance probability is rather similar cross-nationally. Kauppinen et al. [16] investigate the likelihood of social assistance receipt among young adults in Finland, Norway, and Sweden using register data for the birth cohorts 1978–1984. Parental social assistance receipt is again found to be a strong ‘predictor’ in all three countries, and moving out of the parental home early is an important antecedent as well.

Of more methodological relevance, Bhuller, Brinch, & Königs [4] use Norwegian monthly register data to scrutinize the assumptions underlying the Markov process, which is cornerstone for the dynamic discrete choice models often used in the above-mentioned literature. The study concludes that “(..) the standard dynamic random-effects probit model is misspecified, and not suited for an analysis of structural state dependence in welfare benefit receipt (..)” (p. 1869). Thus, trajectories of social assistance receipt are apparently considerably more complicated than what econometric models usually allow for, perhaps indicative of randomness playing a non-trivial role for such benefit utilization processes.

Summing up, several sociodemographic factors (e.g., education and immigrant background) are associated with social assistance probability. What is lacking in the existing literature, however, is *explanatory variables* considered crucial for an improved understanding of social assistance dynamics. Examples comprise friends and family ties, childhood disadvantages (e.g., sexual abuse and bullying), psychological resources such as work motivation, etc. A strength of the present study is our access to such information, which is unobservable in administrative registers. Before the data material is described, we proceed by sketching out the theoretical framework that will serve as a background.

## Theoretical perspectives

### Five sets of explanatory variables

This paper is quite data-driven in the sense that we try to make use of the rich information available in the linked survey-register data material at our disposal. Obviously, there are constraints here imposed by the questions asked while collecting the survey data in 2005, but the data material encompasses a variety of interesting information. Next, we will describe briefly each of the five sets of explanatory variables examined in the empirical analyses: (a) childhood disadvantages, (b) health status, (c) health behaviors, (d) psychological resources, and (e) social ties.

First, *childhood disadvantages* are of potential importance for social assistance dynamics [17]. Negative experiences and events during childhood–e.g., living in poverty for prolonged periods and/or having parents who struggle with addiction problems–could have a long reach, and possibly affect benefit utilization processes directly. Furthermore, neglect and abuse in childhood might translate into both attention problems in school and risk-seeking behavior, which again could lead to a lack of educational qualifications and subsequent difficulties on the labor market (i.e., indirect effect). There is also a possibility for intergenerational transmission of social disadvantages [18]. Some individuals could, for instance, be genetically predisposed to addiction or behavioral problems, and this vulnerability is probably amplified–and possibly triggered–by exposure to unfortunate circumstances while growing up.

Second, it seems likely that *health status* matters for social assistance recipiency. There is a broad range of empirical studies that shows a robust statistical association between (mental/somatic) health problems and weak labor market attachment [19, 20, 21]. Prospective employers are probably hesitant towards hiring someone with a health impairment due to worries about productivity, high sickness absence, and a possible further health deterioration [22: 462–463]. In addition, people with serious health conditions might self-select out of employment altogether, simply because their health status is incompatible with holding wage labor. Disability benefit utilization is a viable option for some health conditions. However, in other cases it could prove more difficult to arrive at a clear diagnosis (e.g., because of multi-morbidity or illnesses that manifest in ‘blurry’ ways), which might imply a higher probability of returning to (or remaining on) social assistance.

Third, *health behaviors* could also be associated with social assistance receipt. It is probably safe to assume that a lifestyle consisting of regular exercise and a varied diet is beneficial for health, but it might seem more counterintuitive to expect it to have a direct impact on benefit utilization processes. Having a healthy lifestyle could, however, prove to be an asset while trying to exit from social assistance, if e.g. employers interpret an athletic body as a signal of conscientiousness and determination. Even more consequential for social assistance careers are probably behaviors such as high alcohol consumption and/or drug problems [23, 24]. Alcohol/drug addiction can be difficult to reconcile with employment, possibly implying longer or multiple spells of SA.

Fourth, *psychological resources* could play a role for social assistance careers. Personal characteristics such as confidence, self-worth and motivation are probably essential cogs in the explanatory wheel for why some people excel, whereas others fail in the lottery of life [25, 26]. Long-term social assistance recipients tend to have an accumulation of health and welfare problems, and a brighter future and economic self-sufficiency could therefore seem farfetched. However, people with a more positive self-image and/or higher work motivation might be more likely to exit permanently from social assistance, for example because of better coping mechanisms while facing (repeated) disappointments during hiring processes. It is common to experience failures while applying for jobs, but the (potential) negative impact of rejections on wellbeing might depend heavily on the individuals’ psychological resources.

Fifth and finally, *social ties* might prove to be of importance for social assistance dynamics [12, 27]. Social ties could in fact be particularly important because this ‘vulnerable group’ tends to have few other resources to rely on. With low levels of educational qualifications and limited work experience (on average), long-term social assistance recipients are often in need of an ‘alternative route’ into employment. To know someone (who knows someone) could be one such route that improves his/her employment prospects. Conversely, social assistance recipients who have a limited number of friends, family members and acquaintances in their network are possibly less likely to reach financial self-sufficiency.

### Labor markets and employment offices

This paper is primarily an empirical assessment of the ‘potency’ of numerous individual-level predictors. However, there are important contextual factors that needs to be acknowledged while interpreting the statistical associations (or lack thereof) between these individual-level predictors and social assistance recipiency. Two such factors are of particular importance: (i) regional/local labor markets, and (ii) municipal Employment offices.

Firstly, the local/regional labor market is consequential because high *labor demand* probably implies more employment opportunities (and job offers) for long-term social assistance recipients. The *industrial structure* could matter as well, for instance whether there are limited or many low-skill, entry-level jobs available for social assistance recipients with few educational credentials. Furthermore, the *employers* in the local/regional area play a key role in deciding whom to hire. Only employers are in a position to take a chance on someone with noticeable ‘scars’ on their CV [28], which long-term social assistance recipients tend to have. Consequently, some recipients could be fortunate enough to get in touch with an employer who is supporting and understanding, and this lucky break could ‘kick-start’ the process of gaining a firm attachment to the labor market.

Secondly, the municipal Employment offices are probably very important for social assistance dynamics [29]. For example, to have a *caseworker* with whom it is easy to communicate and collaborate can ease the transition from welfare reliance to financial self-sufficiency, and such a ‘random’ event of caseworker assignment could therefore have a non-trivial impact on long-term social assistance recipients’ life course. More generally, the *resources* available at the Employment office could also prove to be influential. Tight budgetary constraints in the municipality could e.g., imply that long-term social assistance recipients are deprived of programs and income maintenance schemes that are available in other municipalities.

Unfortunately, we cannot observe the potentially crucial role played by labor markets/employers and Employment offices/caseworkers in the current study because we do not possess this information, but we can examine the importance of 28 individual-level predictors. Of course, other explanatory variables (such as personality and cognitive abilities) could be equally–or more–important for social assistance dynamics. However, we have to make do with the available information in our dataset, the details of which we turn to now.

## Materials and methods

The current study uses a linked survey—register data material, where we follow a cohort of long-term recipients of social assistance in Norway from 2005–2013. *Long-term* is defined as having social assistance as main income source for at least 6 months during the calendar year 2004. In 2005, 562 long-term social assistance recipients responded to a tailor-made living conditions survey. The survey was carried out in close collaboration with the local social assistance administrations in each of the 14 participating Norwegian municipalities. The project management mailed the questionnaires to the local administrations, and they forwarded the survey to social assistance recipients who met the inclusion criteria. The respondents returned the completed questionnaire directly to the project management by mail. A total of four reminders were issued, and the data collection lasted for 9 months. Written informed consent was collected from all respondents who participated in the 2005 survey, and the participants were given the option to refuse the linkage of survey data to administrative register data retrospectively. In a follow-up project, approved by the Norwegian Data Protection Authorities, we got permission to link register data prospectively (i.e., from 2005–2013) for all participants who consented to being included when they were contacted and informed about the follow-up project by Statistics Norway.

The response rate in the 2005 survey (N = 562) was 52.7 in the 14 municipalities, and non-response bias is therefore a concern. However, an empirical assessment showed no significant difference between the participants and non-participants on key individual characteristics assembled from administrative register data (using the ‘FD-Trygd’ database) [30]. Yet, there could still be differences on unobservable characteristics that are of importance for social assistance dynamics, se we need to be cautious while interpreting the findings from the following analyses.

We have linked information derived from Norwegian administrative registers to the survey sample via national identity numbers (in anonymized form, kept by Statistics Norway), enabling us to follow the benefit and employment ‘careers’ for this cohort. We were not able to link information for everyone, and the sample size is therefore quite low (N = 456). On the bright side, the people we were unable to link do not differ systematically on individual characteristics from the individuals included in the linked survey-register sample (S1 Table). Thus, the information is most likely representative for the 2005 cohort, although uncertainty remains as to the generalizability to other municipalities in Norway than the 14 participating ones. It is also important to stress that the main bulk of social assistance spells are rather short in the Nordic countries [6, 7, 14]. Thus, the empirical findings cannot be generalized to Norwegian social assistance recipients as a whole, and are only valid for the more ‘selected’ group of long-term recipients (i.e., social assistance as main income source for minimum 6 months during a year).

### Operationalization

#### Outcome measures

Social assistance is an individual right in Norway, and people may be eligible even if they live with their parents or a partner. However, spouses have a duty to provide financially for each other, and for married individuals, the family/household is the relevant unit while deciding whether he/she is eligible for social assistance or not. This practice is contrasted by the USA, in which the household is the basis for means-testing, regardless of marital status.

The outcome measure is receipt of *social assistance* (SA), derived from the tax register data. Information is available for 2005–2013, and we use the panel structure by specifying three dichotomous versions of the outcome. First, whether a person received any SA in 2013, the last observational year (denoted *SA 2013*). Second, we use the Norwegian National Insurance scheme basic amount (henceforth: *G*), and measure whether a person received more than 1.5G SA (roughly 12 500 € in 2013) at least once during 2005–2013 (denoted *1*.*5G SA ever*). G is regulated by Parliament May 1 every year, and is used as a basis for calculation of benefits and pensions. G is adjusted annually for wage developments and (increasing) costs of living, and therefore well suited for analyses of income data over time. Third, whether the person received some SA every year during the period 2009–2013 (denoted *SA alw*ays).

SA in 2013 is used because, first, we wish to be as up-to-date as possible, and second, because this is an indicator of “sticky” welfare problems. The sample consists of people who were long-term social assistance recipients in 2004, implying that respondents who receive (some) social assistance in 2013 have been in the Norwegian welfare system for ten years, at least. Furthermore, we chose 1.5G because this is a common income threshold in Norway used e.g. for eligibility criteria for unemployment benefits [31]. People who receive at least 1.5G SA during a year will normally have social assistance as their most important income source. Social assistance was a *supplementary* income source for roughly 60 percent of all 120 775 Norwegian recipients in 2013 [32], and one could therefore argue that it is particularly interesting to analyze instances where SA is the *main* income source. Finally, receipt of SA continuously for five years 2009–2013 is an indicator of enduring difficulties with financial self-sufficiency. The prevalence is 31.65, 37.91, and 12.85 for *SA 2013*, *1*.*5G SA ever*, and *SA always*, respectively (see S2 Table). The correlation between the outcome measures is quite strong for *SA always*—*SA 2013* (Pearson’s correlation coefficient (R) = 0.597), moderate for *SA always*–*1*.*5G SA ever* (R = 0.317), and rather weak for *SA 2013*–*1*.*5G SA ever* (R = 0.209) [33]. Thus, the three outcomes are apparently measuring somewhat differing aspects of general social assistance dynamics. Note that none of the three outcome measures explicitly taps into the length of receipt (i.e., number of months), although those who receive more than 1.5G during a calendar year will, in most cases, have been SA recipients for large parts of the year.

Three additional outcome measures are analyzed to compare the empirical findings established for social assistance. First, respondents who earn more than 3G wage income in 2013 are considered to be in *employment*. The 3G threshold is roughly equivalent to what an unskilled worker in a full-time, low paid job earns yearly. Second, *work assessment allowance* is a temporary (max. four years) health-related benefit. To be eligible for work assessment allowance, work capacity must be impaired due to illness or injury by at least 50 percent [34]. The overarching goal is to improve the recipients’ labor market prospects through treatment, employment training and/or courses at the local Employment office. Third, *disability benefit* utilization represents, in most cases, a permanent withdrawal from the labor market due to long-term, serious health problems. Consequently, the empirical findings established for social assistance, a means-tested, temporary income maintenance scheme, will be compared with outcomes representing labor market *success* (employment), labor market *preparation* (work assessment allowance), and labor market *withdrawal* (disability benefit).

#### Explanatory variables and covariates

Turning to the explanatory variables, we have access to six indicators on (a) *childhood disadvantages*. Dichotomous measures (yes = 1;no = 0) for these events during childhood (i.e., before the age of 16) are included: experiences of *economic hardships*, *parental drug/alcohol problem*, *sexual abuse*, *bullying (long-term)*, *attention problems at school*, and *moving*. These events could be considered as exogenous because, first, they all happen before social assistance reliance in time, and second, because these negative events are not chosen by the individual him-/herself. Response bias (e.g., that some people exaggerates their childhood difficulties as a way to rationalize current-day problems) could still be a concern, though.

Six variables on (b) *health status* is used. The 10-item version of Hopkins Symptom Checklist (HSCL-10) is an index varying from 1–4, consisting of the average responses to 10 questions (see [43] for more detailed operationalization of indexes). *HSCL-10* measures symptoms of anxiety and depression [35], and the Cronbach’s alpha in our sample is 0.910. *Psychological wellbeing* is an index consisting of the average answer to three questions on feeling calm, energized and blue (Cronbach’s alpha = 0.733) that ranges from 1–6. *Pain often* is a dichotomous measure (1 = yes;0 = no) on whether the respondent experiences pain often or not. People reporting excellent or very good self-rated health (SRH) are coded 1 (else = 0) on *excellent/very good SRH*. Those who report to be limited (a lot or a little) by their health status while performing simple tasks such as vacuuming or gardening are coded 1 (else = 0) on *limiting illness*. *Physical health issue* is a dichotomous variable (yes = 1;no = 0) measuring whether people have accomplished less than they intended during the last 4 weeks because of their physical health.

Five dichotomous variables on (c) *health behavior* is included. People who report to consume alcohol daily or 2–3 times a week are coded 1 (weekly, 2–3 times a month, monthly, seldom, not in the last year, never = 0) on *drinks often*. Respondents who answer affirmative to whether they currently have a problem with alcohol are coded 1 (else = 0) on *alcohol problem*. A similar coding applies to *drug problem* (current problem = 1, else = 0). People who report to exercise outdoors (e.g., hiking, skiing, cycling) on a daily or weekly basis are coded 1 (monthly, yearly, seldom, never = 0) on *regular exercise (outdoors)*. A similar coding is used for *regular exercise (indoors)*, such as gym and aerobics.

Four variables are included for (d) *psychological resources*. An average of answers to 7 statements (e.g., “there is not much I can do to change important aspects of my life”) make up the index for *mastery* (range = 1–5) [36], for which the Cronbach’s alpha is 0.725 in the current sample. In a similar vein, replies to five statements (e.g., “I feel useless at times”) make up the *self-worth* index (range = 1–4) [37], with an Cronbach’s alpha of 0.804. *Life satisfaction* is measured with the question “How satisfied or dissatisfied are you overall with your life nowadays?”, answers ranging from “very dissatisfied” (1) to “very satisfied” (5). Replies to six statements (e.g., “I would be easily bored if I did not have a job to perform”) make up the *work motivation* index (range = 1–5, Cronbach’s alpha = 0.885). Mastery and self-worth are coded negatively (i.e., higher values, lower self-esteem), whereas life satisfaction and work motivation are coded positively (i.e., higher values, higher satisfaction/motivation).

Seven variables are used for (e) *social ties*. Three questions on generalized trust, honesty and helpfulness comprise the *social capital* index, varying from 0–10 (Cronbach’s alpha = 0.835). People who report to be visited or visit others sometimes a year, seldom or never are coded 1 (daily, weekly, monthly = 0) on *seldom visits/visited*. Those who often feel lonely are coded 1 (sometimes, seldom, never = 0) on *often lonely*. People who state that they have no good friends around (apart from family) are coded 1 (else = 0) on *no close friends around*. Respondents that meets friends sometimes a year or less often than yearly are coded 1 (monthly, weekly, daily = 0) on *seldom meets friends*. A similar coding is applied to *seldom meets siblings* and *seldom meets parents* as well, i.e., less often than monthly encounters are coded 1 (else = 0).

Lastly, basic sociodemographic covariates are included in all model specifications: *Age* (in years) and *age squared*, *gender* (1 = female; 0 = male), *married/cohabiting* (1 = yes; 0 = no), completion of upper secondary or higher *education* (1 = yes; 0 = no), and *country background* (1 = born abroad; 0 = born in Norway). The Pearson’s R is quite weak for these covariates (the highest Pearson’s R is for *married/cohabiting*—*born abroad*: 0.208), implying that collinearity is not a major issue. The next section sketches out the analytical framework.

### Analytical strategy

All outcome measures are dichotomous, but we prefer linear probability models (OLS) throughout for two reasons. First, it is difficult to compare coefficients (and effect sizes) across different samples and model specifications in non-linear models such as logit and probit [38, 39]. Second, the results from OLS are easier to interpret (i.e., percentage point differences) compared to e.g., odds ratios [40]. To examine whether breach of functional form assumption is consequential for the empirical results, we will estimate the same models with logistic regression as well. By using average marginal effects (AME), we can compare the coefficients from linear and logistic regression and see to what extent they differ. Note that this study is only able to show statistical associations, and no causal connections can be established with the current data and analytical approach.

The analysis proceeds in three steps. In step one, we run linear probability models of social assistance receipt, including one explanatory variable at a time, adjusting only for basic sociodemographic covariates (age, gender, marital status, education, and country background). We repeat this for all 28 explanatory variables, which amounts to 84 separate regressions since we use three outcome measures. It is important to consider potential problems with multi-collinearity [41], and especially so when the number of observations is rather low. The correlation for several of the included explanatory variables is quite strong (0.479 for *pain often*—*limiting illness* and 0.601 for *mastery*—*self-worth*, for example), which is why parsimonious model specifications are preferred in the first step. Only the coefficient for the explanatory variable(s) is shown in the table due to space restrictions.

In step two, we include the most ‘powerful predictors’–defined as *consistent association and substantial effect size–*established in step one in the same model specification. An explanatory variable is considered to be a ‘powerful predictor’ if two conditions are met. First, the variable is significantly associated (on the 95 percent level) with at least two of the three outcome measures (i.e., *consistent association*). If an explanatory variable is significantly associated with only one outcome measure, it might be due to chance. This condition is especially important in the current study due to the large number of explanatory variables (N = 28). Second, the estimated coefficient is reasonably large (i.e., *substantial effect size*). For a dichotomous explanatory variable, we consider a 6–7 percentage point difference to be the lower limit. It is more complicated for continuous variables, because we have to consider the variation in respondents’ scores. The full range for *work motivation* (std.dev = 1.135), for example, is 1–6, but only 9.95 percent score lower than 3. Thus, an increase on the work motivation scale of more than two points is empirically unrealistic. Similar reasoning applies to *social capital* (std.dev = 2.198) that varies from 0–10, while ¾ of all respondents score between 2 and 7. Obviously, these two conditions–*consistent associations* and *substantial effect size*–are fairly arbitrary, but we believe there is some merit to the path chosen here.

The *explicit comparison* across different outcome measures is introduced in step three, where we repeat step one for *employment*, *work assessment allowance*, and *disability benefit*, (i.e., 84 additional regressions). By examining whether the same or other individual-level predictors are of importance, we hope to come a few steps closer in explaining why some people are unable to reach financial self-sufficiency. For instance, it seems reasonable to expect that poor (somatic) health is a more powerful predictor of disability benefit utilization than social assistance recipiency. Similarly, it will not come as a surprise if individuals who experienced childhood disadvantages are more likely to be recipients of social assistance than a health-related benefit such as work assessment allowance. More generally, the comparison enables us to see whether differences in income source characteristics (see Table 1) translate into varying ‘explanatory power’ for the 28 indicators. Importantly, social assistance differs from both work assessment allowance and disability benefit because social assistance is (i) means-tested, (ii) not a health-related benefit, (iii) a short-term economic support, (iv) granted by caseworkers’ decision only, and (v) because SA is funded by the municipality (as opposed to the State). These nuances could prove to be of importance for the interpretation of results.

**Table 1:** Summary of income source characteristics.

Income source	Social assistance	Employment	Work assessment allowance	Disability benefit
Permanency	Temporary	-	Temporary	Permanent
Duration	Short-term	-	Long-term (max. 4 years)	-
Means-tested?	Yes	-	No	No
Health-related?	No	-	Yes	Yes
Decision-maker	Caseworker	Employer	Caseworker & doctor	Caseworker & doctor
Funding	Municipality	Firm	State	State

## Results

### Descriptive statistics

Descriptive statistics for the five sets of explanatory variables (N = 28) is shown in S3 Table. There is an accumulation of disadvantages and health problems in the current sample. For example, 43.43 percent experienced *economic hardships* during childhood, 58.60 percent report *attention problems* in school, long-lasting *bullying* is reported by one-third of the sample, 48.43 percent have *chronic pain*, and almost one-fourth report *drug problems*. We cannot compare all of these numbers with the population as a whole in the current paper, but previous research on the current social assistance cohort indicates that the sample is indeed characterized by an overrepresentation of various difficulties and challenges [42, 43]. Moreover, a comparison with the general population is possible for the childhood disadvantage variables (S4 Table). The prevalence of *attention problems* is only 11.79 percent for the population as a whole, compared to almost 6/10 of long-term social assistance recipients. *Sexual abuse* is also much more common among the 2005 social assistance cohort: 14.42 percent vs. 2.79 percent in the general population. Similar overrepresentation is found for all six childhood disadvantages.

### Social assistance dynamics

Table 2 shows the results from a series of OLS regressions with social assistance receipt (SA 2013; 1.5G SA ever; SA always) as the outcome, and the 28 explanatory variables included separately alongside sociodemographic covariates (age, gender, marital status, education, and country background). The perhaps most striking finding from these regressions is the *lack of* statistically significant associations: very few of the explanatory variables are able to ‘predict’ social assistance receipt. This could be due to the low number of observations, but the coefficients are quite small in several cases (see e.g., *parental drug/alcohol problem*, *pain often*, and *no close friends around*). Furthermore, some of the coefficients change sign across the different outcome measures (see e.g., *bullying*, *limiting illness*, and *physical health issue*), clearly showing that low statistical power is not the sole explanation for why so few variables are statistically significant.

**Table 2:** Linear probability model (OLS) of social assistance, by (a) childhood disadvantages, (b) health status, (c) health behavior, (d) psychological resources, or (e) social ties, and covariates.

	Outcome: social assistance (SA)		
Explanatory variable	(1) SA in 2013	(2) 1.5G SA Ever (2005–2013)	(3) SA Always (2009–2013)
**(A) Childhood disadvantages**			
Economic hardships	0.052 (0.049)	0.011 (0.049)	0.032 (0.034)
Parental drug/alcohol problem	0.014 (0.054)	0.010 (0.054)	-0.032 (0.036)
Sexual abuse	-0.026 (0.074)	0.115 (0.073)	-0.027 (0.050)
Bullying (long-term)	0.073 (0.053)	-0.009 (0.053)	0.020 (0.037)
Attention problems school	0.049 (0.054)	0.089* (0.053)	-0.025 (0.037)
Moving	0.041 (0.051)	-0.043 (0.051)	-0.011 (0.035)
**(B) Health status**			
HSCL-10	0.031 (0.034)	0.108*** (0.033)	0.033 (0.023)
Psychological wellbeing	-0.031 (0.021)	-0.027 (0.021)	-0.010 (0.014)
Pain often	0.018 (0.050)	0.017 (0.049)	-0.014 (0.034)
Excellent/very good SRH	0.005 (0.058)	-0.015 (0.058)	-0.011 (0.040)
Limiting illness	-0.064 (0.049)	0.059 (0.048)	0.013 (0.033)
Physical health issue	0.010 (0.049)	-0.023 (0.049)	0.009 (0.034)
**(C) Health behaviors**			
Drinks often	0.018 (0.064)	0.101* (0.061)	0.021 (0.042)
Alcohol problem	0.115* (0.069)	0.113* (0.065)	0.020 (0.045)
Drug problem	0.274*** (0.056)	0.279*** (0.055)	0.161*** (0.038)
Regular exercise (outdoors)	0.044 (0.048)	0.067 (0.047)	0.029 (0.033)
Regular exercise (indoors)	0.031 (0.062)	-0.001 (0.062)	0.015 (0.043)
**(D) Psychological resources**			
Mastery	0.022 (0.032)	0.069** (0.031)	0.016 (0.021)
Self-worth	0.046 (0.036)	0.059* (0.035)	0.012 (0.024)
Life satisfaction	-0.032 (0.022)	-0.046** (0.021)	-0.013 (0.015)
Work motivation	-0.047* (0.024)	-0.048** (0.024)	-0.032** (0.016)
**(E) Social ties**			
Social capital	-0.025** (0.011)	-0.039*** (0.011)	-0.011 (0.008)
Seldom visits/visited	0.136** (0.057)	-0.077 (0.056)	0.034 (0.039)
Often lonely	0.112** (0.050)	0.098* (0.050)	0.073** (0.035)
No close friends around	0.027 (0.055)	0.011 (0.054)	0.006 (0.037)
Seldom meets friends	-0.062 (0.061)	-0.063 (0.060)	-0.012 (0.042)
Seldom meets siblings	-0.038 (0.053)	-0.019 (0.051)	-0.020 (0.036)
Seldom meets parents	-0.025 (0.057)	-0.061 (0.057)	-0.061 (0.040)

Significance level:

*** = 0.01

** = 0.05

* = 0.1; Standard errors in parentheses; Only the coefficient for the explanatory variable(s) shown; The explanatory variables are included separately; All models controls for age (and age^2^), female, married/cohab., VGS/higher educ., and born abroad.

A couple of important exceptions do appear, however. Firstly, people with self-reported *drug problem* have a much higher probability of social assistance in all three models. The effect size is very large: 16–28 percentage points. Secondly, the *work motivation* index is consistently associated with social assistance, and the effect size is considerable: An increase from e.g., 3 to 5 implies between 6.4 (3.2*2) and 9.6 (4.8*2) percentage points lower social assistance probability. Thirdly, those who report to be *often lonely* have a higher social assistance probability. The effect size is yet again substantial, varying from 7.3 to 11.2 percentage points. All models have been estimated with logistic regression to examine whether breach of functional form assumption is consequential. Average marginal effects (AME) are shown in S5 Table, and the empirical findings are strikingly similar.

Several other explanatory variables are associated with social assistance, but not in a consistent and substantial manner. For example, those who *drink often* and report *alcohol problem* have roughly 10–11 percentage point higher social assistance likelihood in 3/6 possible model specifications. Further, *mastery* (b = 0.069), *self-worth* (b = 0.059), and *life satisfaction* (b = -0.046) are all associated with probability of 1.5G SA during 2005–2013 (and the effect sizes are substantial), but not for the two remaining outcomes. *Social capital* is significant in two models, but the effect size is quite small for *SA 2013* (b = -0.025). *Seldom visits/visited* is associated with a 13.6 percentage point higher likelihood of SA in 2013, but the coefficient is negative and rather large for SA 1.5 G (b = -0.077).

Table 3 shows the results from more extended linear probability models, where *drug problem*, *work motivation* and *often lonely* are included, in addition to the sociodemographic covariates. The highest Pearson’s R is 0.208 (for *married/cohabiting*—*born abroad*), implying that multi-collinearity is not a big problem (see notes below Table 3). The coefficients are shown for the sociodemographic covariates as well, and the first thing to highlight is the consistent and substantial association between *country background* and social assistance. Long-term recipients of social assistance who are born outside Norway have a 13–18 percentage point higher likelihood of social assistance receipt. The ‘predictive power’ of self-reported *drug problem* is still remarkable, with an effect size varying from 14.1 to 27.8 percentage points. *Work motivation* is significant (on the 90 percent level) only for *SA always*, but the effect size (b = -0.028) is moderate/small. Lastly, those who are *often lonely* do not differ significantly in the extended model, and the effect size is quite small (5.5–5.6 percentage points) for two of the outcome measures.

**Table 3:** Linear probability model (OLS) of social assistance, by age (and age squared), gender, marital status, education, country background, drug problem, work motivation, and often lonely.

	Outcome: social assistance (SA)		
	(1) SA in 2013	(2) 1.5G SA Ever (2005–2013)	(3) SA Always (2009–2013)
Constant	0.269 (0.276)	-0.090 (0.273)	0.198 (0.188)
Age	0.003 (0.016)	0.024 (0.016)	-0.004 (0.011)
Age^2^	-0.000 (0.000)	-0.000 (0.000)	0.000 (0.000)
Female	-0.002 (0.049)	-0.061 (0.049)	0.010 (0.034)
Married/cohab.	0.010 (0.061)	-0.015 (0.061)	0.001 (0.042)
VGS/higher educ.	-0.005 (0.064)	0.010 (0.063)	-0.032 (0.043)
Born abroad	0.160** (0.072)	0.181*** (0.069)	0.129*** (0.047)
Drug problem	0.232*** (0.059)	0.278*** (0.058)	0.141*** (0.040)
Work motivation	-0.035 (0.024)	-0.031 (0.024)	-0.028* (0.016)
Often lonely	0.071 (0.051)	0.056 (0.050)	0.055 (0.035)
Adjusted R^2^	0.050	0.096	0.046
N	363	392	392

Significance level:

*** = 0.01 ** = 0.05 * = 0.1; Standard errors in parentheses; Pearson’s correlation coefficient is above 0.150 in eight cases: (i) age—VGS/higher educ. = 0.160***, (ii) VGS/higher educ.—married/cohab. = 0.188***, (iii) born abroad—married/cohab. = 0.208***, (iv) drug problem—female = -0.164***, (v) drug problem—born abroad = -0.179***, (vi) drug problem—work motivation = -0.192***, often lonely—married/cohab = -0.174***, and often lonely—drug problem = 0.154***.

Our main finding thus far is the *lack of* statistically significant associations between (a) childhood disadvantages, (b) health status, (c) health behavior, (d) psychological resources, and (e) social ties and social assistance probability. Nevertheless, two important exceptions do appear. Being *born abroad* and having a *drug problem* are associated with social assistance receipt in a consistent and substantial manner. The effect size for both of these explanatory variables is large, with a 12.9–18.1 (born abroad) and 14.1–27.8 (drug problem) percentage point higher SA probability (Table 3). There is also some indication that those who are *often lonely* and those with low *work motivation* have a higher likelihood of returning to social assistance. However, the effect sizes are moderate/small for both variables, and none of the coefficients are statistically significant on the 95 percent level, in the extended linear probability models in Table 3.

### Employment, work assessment allowance and disability benefit

Somewhat surprisingly, it proved difficult to ‘statistically predict’ the dynamics of social assistance for a cohort of long-term social assistance recipients in Norway. To see whether social assistance receipt stands out in this regard, we have also run analyses with three additional outcome measures. First, several indicators in all five explanatory variable groups are significantly associated with *employment* likelihood in 2013 (Table 4). Notably, having a *limiting illness* (b = -0.135), experiencing *long-term bullying* (b = -0.133), and reporting *attention problems* in school (-0.099) are apparently damaging for employment prospects. Furthermore, *mastery* (range = 1–5), *self-worth* (range = 1–4), and *work motivation* (range = 1–5) all show a strong statistical association, with an effect size of roughly 7–8 percentage points for each unit increase. Similarly, for each unit increase in *HSCL-10* (range = 1–4), the employment probability decreases with 7 percentage points. Finally, both *social capital* (range = 0–10, b = 0.019) and *often lonely* (b = -0.088) is significantly related with likelihood of earning wage income.

**Table 4:** Linear probability model (OLS) of (1) wage income, (2) work assessment allowance, or (3) disability benefit, by (a) childhood disadvantages, (b) health status, (c) health behavior, (d) psychological resources, or (e) social ties, and covariates.

	Additional outcome measures		
Explanatory variable	(1) Wage income 2013‡	(2) Work assessment allowance 2013	(3) Disability benefit 2013
**(A) Childhood disadvantages**			
Economic hardships	-0.036 (0.043)	0.129*** (0.048)	0.001 (0.045)
Parental drug/alcohol problem	0.023 (0.048)	0.096* (0.053)	-0.026 (0.050)
Sexual abuse	-0.110* (0.065)	0.152** (0.073)	-0.007 (0.066)
Bullying (long-term)	-0.133*** (0.046)	0.095* (0.052)	0.108** (0.048)
Attention problems school	-0.099** (0.047)	0.077 (0.052)	0.046 (0.048)
Moving	-0.046 (0.045)	0.080 (0.050)	-0.008 (0.046)
**(B) Health status**			
HSCL-10	-0.072** (0.029)	0.068** (0.033)	0.107*** (0.030)
Psychological wellbeing	0.039** (0.018)	-0.061*** (0.021)	-0.049*** (0.019)
Pain often	-0.060 (0.043)	-0.006 (0.049)	0.168*** (0.044)
Excellent/very good SRH	0.092* (0.050)	-0.150*** (0.057)	-0.169*** (0.052)
Limiting illness	-0.135*** (0.042)	0.017 (0.049)	0.249*** (0.043)
Physical health issue	-0.008 (0.043)	-0.033 (0.048)	0.142*** (0.044)
**(C) Health behaviors**			
Drinks often	-0.038 (0.056)	0.030 (0.063)	0.021 (0.056)
Alcohol problem	-0.081 (0.060)	0.051 (0.069)	0.116* (0.059)
Drug problem	-0.076 (0.050)	0.017 (0.057)	-0.018 (0.052)
Regular exercise (outdoors)	-0.004 (0.041)	0.063 (0.047)	-0.030 (0.043)
Regular exercise (indoors)	0.096* (0.053)	-0.111* (0.061)	-0.059 (0.056)
**(D) Psychological resources**			
Mastery	-0.083*** (0.027)	0.063** (0.031)	0.069** (0.028)
Self-worth	-0.073** (0.031)	0.069* (0.035)	0.076** (0.033)
Life satisfaction	0.023 (0.019)	-0.033 (0.021)	-0.036* (0.019)
Work motivation	0.068*** (0.019)	-0.001 (0.022)	-0.052*** (0.019)
**(E) Social ties**			
Social capital	0.019** (0.010)	0.005 (0.011)	-0.017* (0.010)
Seldom visits/visited	-0.035 (0.050)	-0.051 (0.056)	0.116** (0.051)
Often lonely	-0.088** (0.044)	0.093* (0.050)	0.046 (0.046)
No close friends around	0.032 (0.048)	-0.024 (0.054)	0.045 (0.049)
Seldom meets friends	0.044 (0.053)	0.001 (0.060)	-0.006 (0.055)
Seldom meets siblings	-0.008 (0.045)	-0.027 (0.052)	0.032 (0.047)
Seldom meets parents	0.024 (0.050)	0.048 (0.058)	0.010 (0.052)

Significance level:

*** = 0.01

** = 0.05

* = 0.1; Standard errors in parentheses; Only the coefficient for the explanatory variable(s) shown; The explanatory variables are included separately; All models controls for age (and age^2^), female, married/cohab., VGS/higher educ., and born abroad

‡Measured with the Norwegian National Insurance scheme basic amount (G). G is adjusted annually for wage developments and costs of living. The threshold is set to 3G, or roughly 25 000 € in 2013.

Second, childhood disadvantages seem to be of importance for the likelihood of receiving *work assessment allowance* in 2013, and the coefficients are especially large for *economic hardships* (b = 0.129) and *sexual abuse* (b = 0.152). In addition, respondents that report *bullying* and *parental drug/alcohol problems* during childhood are at a higher risk of work assessment allowance, with an effect size of roughly 9.5 percentage points. The two mental health indicators–*HSCL-10* and *psychological wellbeing–*also show a strong statistical association. Several other variables are significantly associated with work assessment allowance as well, for example *mastery* (b = 0.063) and *often lonely* (b = 0.093).

Third, all six health indicators are significant predictors of *disability benefit* utilization in 2013. The strongest statistical association is found for *limiting illness*, where the effect size is 25 percentage points, followed by *excellent/very good health* (b = -0.169) and *pain often* (b = 0.168). As expected, the statistical relationships tends to be stronger for the somatic health indicators than for the mental health indicators. Slightly more surprising is the relatively strong effect sizes for *long-term bullying* (b = 0.108) and *seldom visits/visited* (b = 0.116), indicating that social disadvantages–in addition to health reasons–are of potential importance for permanent labor market withdrawal. Finally, psychological resources–*mastery* (b = 0.069); *self-worth* (b = 0.076); and *work motivation* (b = -0.052)–are quite powerful predictors of disability benefit utilization as well.

Thus, numerous explanatory variables are associated with labor market *success* (employment), labor market *preparation* (work assessment allowance), and labor market *withdrawal* (disability benefit). However, when it comes to social assistance, a means-tested, temporary benefit that is supposed to be short-term, the indicators fall short. Next, we proceed to a discussion of the presented results.

## Discussion

### Disentangling the dynamics of social assistance

The aim of the current paper was to disentangle the dynamics of social assistance in Norway. We did so by examining the potential importance of 28 explanatory variables on (a) childhood disadvantages, (b) health status, (c) health behavior, (d) psychological resources, and (e) social ties. Three main findings appear. First, it proved very challenging to ‘predict’ statistically which people who will return to social assistance. Merely 3/28 explanatory variables were associated with social assistance in a consistent and substantial manner. When more extended model specifications were estimated, the associations were attenuated considerably for two of them (*work motivation* and *often lonely*). The 28 indicators do a far better job in ‘predicting’ both labor market *success* (employment), labor market *preparation* (work assessment allowance), and labor market *withdrawal* (disability benefit). Thus, it seems that while employment on the one hand, and health-related benefits on the other, can be seen as substitutes (often gaining significant coefficients with opposite signs), social assistance receipt seems to be driven by a separate set of factors largely unobserved by our study.

The lack of significant explanatory variables could indicate that there is a non-negligible component of *randomness* involved in social assistance dynamics. Some long-term social assistance recipients are perhaps fortunate while being assigned a caseworker. Having a caseworker at the local Employment Office with whom they are able to communicate and collaborate in a streamlined fashion can ease the transition from social assistance reliance to financial self-sufficiency. Similarly, getting in touch with an employer who is supporting and understanding could ‘kick-start’ the process of gaining a firmer labor market attachment. Thus, these more or less random events may have a non-trivial impact on how well/poor a long-term social assistance recipient fares over the life course, and these interplays are difficult to observe empirically with linked survey-register data. This could explain why so few of the 28 explanatory variables were able to ‘predict’ social assistance dynamics. One could argue that the study by Bhuller, Brinch, & Königs [4], which underline that social assistance receipt is considerably more complex than what econometric models usually allow for, supports this ‘randomness’ interpretation. We have to stress, however, that these non-significant findings might not generalize to other geographical areas than the 14 participating Norwegian municipalities. Furthermore, the relatively small sample size implies that the current study is sensitive to error(s) stemming from both response bias (e.g., exaggeration of childhood difficulties) and non-response bias (i.e., non-participants could be worse off on (un-)observable characteristics than the survey participants). It is also conceivable that the 28 explanatory variables would have done a better job in predicting social assistance receipt of a very long-term kind (e.g., social assistance receipt for more than 12 consecutive months).

The explanatory variables are better ‘predictors’ of employment, work assessment allowance and disability benefit, which is yet another indication that randomness could play a non-negligible role for social assistance recipiency. Social assistance is supposed to be a short-term financial support [1], implying that caseworkers perhaps feel pressured, both from the recipient and from his/her superiors, to look for a more permanent income source. This could help explain the somewhat counterintuitive finding that childhood disadvantages are associated with a health-related benefit such as work assessment allowance, but not with social assistance. Generally, caseworkers could be inclined to enroll people in State-funded programs (c.f. Table 1), for example due to tight municipal budgetary constraints. To opt for work assessment allowance is probably positive for many recipients because, first, it is a more long-term income source, and second, because labor market prospects could improve through the treatment, employment training and/or courses provided [34]. Moreover, the descriptive statistics (S3 Table) clearly shows that this social assistance cohort have poor health on average, and there could therefore be good reasons why ‘social’ disadvantages are associated with a higher likelihood of health-related benefits.

*Signaling* theory invokes a slightly different interpretation of the non-significant associations established for social assistance [44]. The presented results show that *differences* within this cohort of SA recipients in terms of problems and resources are unable to ‘predict’ future social assistance dynamics. Yet, the individuals included in the current study also have one important thing *in common*, namely that they have all been long-term recipients of social assistance. This will manifest as noticeable “gaps” in the résumé, and potential employers will probably interpret this as a signal of potential risk [28]. Risk-averse employers will therefore be skeptical to hire such candidates regardless of the resources that he or she may possess. Consequently, the result could be prolonged difficulties on the labor market, and continued need of financial support from the welfare state apparatus.

A second major finding in the current study is that those with a self-reported *drug problem* have a much higher probability of returning to social assistance, the effect size varying from 13.5–27.2 percentage points. The prevalence for *SA 2013*, *1*.*5G SA ever*, and *SA always* is 31.65, 37.91, and 12.85, respectively, and drug problem is thus able to explain–in a statistical sense–a large bulk of the variation. A drug problem will often be very difficult to reconcile with employment. Individuals with drug problems are often in need of help and support from the social welfare system, but the possible tools are quite limited. For people with serious somatic and mental health impairments, disability benefit is an alternative that can reduce economic uncertainties in a more permanent manner. However, disability benefit is often not considered as a viable option for people with drug problems because addiction is not viewed as a legitimate disability [45]. Caseworkers therefore have fewer possibilities, implying that a return to social assistance is more likely. Note that there is some indication that alcohol problems is associated with risk of social assistance receipt as well (see Table 2), although in a less consistent manner. Thus, more attention to addiction issues in general is warranted in order to improve quality of life, and possibly increase labor market participation, among long-term recipients of social assistance.

Third, being *born abroad* also proved to be a very important social assistance ‘predictor’. Foreign-born long-term social assistance recipients have a heightened probability of returning to social assistance, and the effect size is almost on par with drug problems; 13.1–18.2 percentage points in the extended model specification (Table 3). Social assistance recipients with immigrant background are most likely disadvantages as to language skills, educational credentials (e.g., their education not being recognized by the Norwegian Agency for Quality Assurance in Education), and previous labor market experience. Thus, it is more difficult for social assistance recipients with immigrant background to gain employment in competition with other job applicants that, on average, possess credentials and work experience considered more relevant in the Norwegian context. Furthermore, there is firm evidence that people with non-native sounding names are discriminated in recruitment processes in Norway [46, 47]. Consequently, the barriers are considerably higher for access to financial self-sufficiency for recipients with immigrant background. Political action is needed to combat labor market discrimination, where e.g., anonymization of names in the first phase of recruitment processes could be one policy solution that is worth trialing.

In summary, it is rather difficult to ‘predict’ which people who will return to social assistance, with two crucial exceptions: having a *drug problem* and being *born abroad*. Supplementary analyses with employment, disability benefit, and work assessment allowance as the outcome measure (see Table 4) tell a different tale, where a significant and powerful association was established for several of the 28 indicators. Thus, the explanatory variables are apparently decent predictors of both labor market *success*, labor market *preparation*, and labor market *withdrawal*, but they do a considerably worse job while analyzing social assistance receipt. This could be interpreted in accordance with the above-mentioned randomness explanation. Uncertainties remain, however, and more research is needed in order to disentangle further the dynamics of social assistance. Similar studies as the current one with a larger sample size and several survey waves will probably be highly informative. Qualitative studies where social assistance recipients are followed closely over time–e.g., during meetings and consultations with caseworkers, (prospective) employers, and health professionals–could also reveal important insights.

### Strengths and limitations

We end by emphasizing some of the most important strengths and limitations. The current study uses a linked survey—register data material that includes a broad range of information that is normally unavailable for researchers. With the 2005 survey data, it is possible to examine the importance of e.g. childhood disadvantages, psychological resources, and (lack of) family and friends. The register data for 2005–2013 provides access to high-quality information on benefits and employment with low measurement error and where non-response is not a concern. Thus, we are able to add to the existing literature on social assistance dynamics by investigating individual characteristics that are ‘unobserved heterogeneity’ in studies relying on administrative data sources only. In addition, the examined childhood disadvantages may be exogenous because, first, the temporal order is clear (i.e., the events precedes social assistance receipt), and second, the individuals are not responsible for these negative events themselves. However, (non-)response bias is still a concern, and no causal connections can be established in this study.

There are other important caveats to consider as well. First, a low number of observations implies a loss of statistical power. Second, the explanatory variables were measured back in 2005, and a lot could have changed since then. For instance, health status can improve/worsen considerably and people can recover from alcohol/drug addiction. Third, the large amount of explanatory variables (N = 28) increases the risk of some of them being significantly associated with social assistance purely by chance. We try to circumvent this problem by only highlighting explanatory variables that are associated with social assistance in a consistent and substantial manner. Fourth, we only possess information on long-term recipients of social assistance in 2004. Thus, we cannot generalize our empirical findings neither to social assistance recipients as a whole, nor to other ‘vulnerable groups’ (such as long-term unemployed). Furthermore, uncertainty remains as to whether the presented results are applicable for other, more recent long-term social assistance cohorts.

Fifth, the overrepresentation of health and welfare problems in the current sample (S3 and S4 Tables) probably explains some of the counterintuitive empirical findings. For instance, educational level is far from statistically significant (Table 3). This does not mean that education is inconsequential for social assistance dynamics in Norway, but is rather a reflection of the ‘selected’ sample. Similarly, the lack of statistical associations between childhood disadvantages and social assistance likelihood does not imply that the examined events (e.g., bullying, parental drug/alcohol problem, sexual abuse) are inconsequential for people’s life course. On the contrary, these disadvantages may have been important ‘trigger events’ for why the respondents became long-term social assistance recipients in the first place. Sixth, other potentially important explanatory variables are omitted from our study, such as personality, cognitive abilities, and biomarkers (although they probably are highly correlated with one or several of the 28 explanatory variables). Seventh, there could be important geospatial labor market elements at play here, but information on municipality were omitted from this study due to privacy concerns. Eight and finally, measurement error (e.g., recall bias) is always a concern while working with survey data.

## Supporting information

S1 Table Descriptive statistics on selected variables for (1) linked survey-register sample and (2) survey respondents without register information. (DOCX)

S2 Table Descriptive statistics on social assistance receipt and covariates (age, gender, marital status, educational level, and country background). (DOCX)

S3 Table Descriptive statistics on five sets of explanatory variables: (a) childhood disadvantages, (b) health status, (c) health behavior, (d) psychological resources, and (e) social ties. Percent (*mean* in italics).
(DOCX)

S4 Table Prevalence of childhood disadvantages, a comparison between (1) long-term social assistance recipients, and (2) the general population. Percent.
(DOCX)

S5 Table Logistic regression analysis of social assistance, by (a) childhood disadvantages, (b) health status, (c) health behavior, (d) psychological resources, or (e) social ties, and covariates (age, age squared, gender, marital status, educational level, and country background). Average marginal effects (AME) shown.
(DOCX)
