# Supplementary material for: Disentangling the dynamics of social assistance: A linked survey—Register data cohort study of long-term social assistance recipients in Norway
Source: PLoS One. 2020 Mar 27;15(3):e0230891. doi: 10.1371/journal.pone.0230891 (PMC7100955; doi:10.1371/journal.pone.0230891)
Supplement: S1 Table — (DOCX) [file pone.0230891.s001.docx]

**S1 Table. Descriptive statistics on selected variables for (1) linked survey-register sample and (2) survey respondents without register information.**

|  | (1)  Register sample | (2)  Missing register information |
| --- | --- | --- |
| Female | 42.27 (N=459) | 42.11 (N=95) |
| Age | 33.66 (N=450) | 34.96 (N=89) |
| Married/cohabiting | 21.35 (N=459) | 24.47 (N=94) |
| Ethnic minority | 19.02 (N=347) | 22.67 (N=75) |
| Upper secondary/higher education | 17.87 (N=442) | 17.39 (N=92) |
| Income (in NOK) | 46 123.17 (N=348) | 79 478.57*** (N=70) |
| Excellent/ very good self-rated health | 21.51 (N=451) | 21.51 (N=93) |
| Limiting illness | 42.38 (N=446) | 48.31 (N=89) |
| Experiences pain often | 48.43 (N=446) | 37.63* (N=93) |

T-test on the differences in means; Significance level: *** = 0.01 ** = 0.05 * = 0.1
