# Supplementary material for: Disentangling the dynamics of social assistance: A linked survey—Register data cohort study of long-term social assistance recipients in Norway
Source: PLoS One. 2020 Mar 27;15(3):e0230891. doi: 10.1371/journal.pone.0230891 (PMC7100955; doi:10.1371/journal.pone.0230891)
Supplement: S2 Table — (DOCX) [file pone.0230891.s002.docx]

**S2 Table. Descriptive statistics on social assistance receipt and covariates (age, gender, marital status, educational level, and country background).**

|  | Percent | N |
| --- | --- | --- |
| **Social assistance** |  |  |
| 2013 yes | 31.65 | 417 |
| 1.5 G ever (2005—2013) | 37.91 | 459 |
| Always (2009—2013) | 12.85 | 459 |
| **Additional outcome measures** |  |  |
| Wage income 2013 | 20.86 | 417 |
| Disability benefit 2013 | 32.03 | 459 |
| Work assessment allowance 2013 | 29.74 | 417 |
| **Covariates** |  |  |
| Age | 33.66 | 450 |
| Female | 42.27 | 459 |
| Married/cohabiting | 21.35 | 459 |
| Upper secondary/higher education | 17.87 | 442 |
| Born abroad | 17.82 | 449 |
