# Supplementary material for: Disentangling the dynamics of social assistance: A linked survey—Register data cohort study of long-term social assistance recipients in Norway
Source: PLoS One. 2020 Mar 27;15(3):e0230891. doi: 10.1371/journal.pone.0230891 (PMC7100955; doi:10.1371/journal.pone.0230891)
Supplement: S3 Table — Descriptive statistics on five sets of explanatory variables: (a) childhood disadvantages, (b) health status, (c) health behavior, (d) psychological resources, and (e) social ties. Percent (mean in italics). (DOCX) [file pone.0230891.s003.docx]

**S3 Table. Descriptive statistics on five sets of explanatory variables: (a) childhood disadvantages, (b) health status, (c) health behavior, (d) psychological resources, and (e) social ties. Percent (mean in italics).**

| Explanatory variables | Percent/mean | N |
| --- | --- | --- |
| **(A) Childhood disadvantages** |  |  |
| Economic hardships | 43.43 | 426 |
| Parental drug/alcohol problem | 28.91 | 422 |
| Sexual abuse | 14.42 | 423 |
| Bullying (long-term) | 32.63 | 429 |
| Attention problems school | 58.60 | 430 |
| Moving | 34.58 | 428 |
| **(B) Health status** |  |  |
| HSCL-10 (index; 1—4) | *2.12* | 406 |
| Psychological wellbeing (index; 1—6) | *3.22* | 421 |
| Pain often | 48.43 | 446 |
| Excellent/very good SRH | 21.51 | 451 |
| Limiting illness | 42.38 | 446 |
| Physical health issue | 49.32 | 442 |
| **(C) Health behaviors** |  |  |
| Drinks often | 19.55 | 440 |
| Alcohol problem | 16.01 | 456 |
| Drug problem | 23.68 | 456 |
| Regular exercise (outdoors) | 52.53 | 455 |
| Regular exercise (indoors) | 19.07 | 451 |
| **(D) Psychological resources** |  |  |
| Mastery (index; 1—5) | *2.82* | 431 |
| Self-worth (index; 1—4) | *2.44* | 427 |
| Life satisfaction (1—5) | *2.81* | 453 |
| Work motivation (index; 1—5) | *3.84* | 432 |
| **(E) Social ties** |  |  |
| Social capital (index; 0—10) | *4.77* | 443 |
| Seldom visits/visited | 23.30 | 455 |
| Often lonely | 34.73 | 452 |
| No close friends around | 26.09 | 456 |
| Seldom meets friends | 20.44 | 450 |
| Seldom meets siblings | 42.42 | 422 |
| Seldom meets parents | 31.57 | 396 |

Cronbach’s alpha: HSCL-10 = 0.910 (10 items), psychological wellbeing = 0.733 (3 items), mastery = 0.725 (7 items), self-worth = 0.804 (5 items), work motivation = 0.881 (6 items), social capital = 0.835 (3 items).
