# Supplementary material for: Disentangling the dynamics of social assistance: A linked survey—Register data cohort study of long-term social assistance recipients in Norway
Source: PLoS One. 2020 Mar 27;15(3):e0230891. doi: 10.1371/journal.pone.0230891 (PMC7100955; doi:10.1371/journal.pone.0230891)
Supplement: S4 Table — Percent. (DOCX) [file pone.0230891.s004.docx]

**S4 Table. Prevalence of childhood disadvantages, a comparison between (1) long-term social assistance recipients, and (2) the general population. Percent.**

| **Explanatory variables – childhood disadvantages** | **(1)**  **Long-term social assistance recipientsa** | **(2)**  **General populationb** |
| --- | --- | --- |
| Economic hardships | 43.43 (N=426) | 23.21 (N=3072) |
| Parental drug/alcohol prob. | 28.91 (N=422) | 11.29 (N=3056) |
| Sexual abuse | 14.42 (N=423) | 2.79 (N=3053) |
| Bullying (long-term) | 32.63 (N=429) | 10.74 (N=3053) |
| Attention problems school | 58.60 (N=430) | 11.79 (N=3053) |
| Moving | 34.58 (N=428) | 16.11 (N=3048) |

^a^ Survey among long-term social assistance recipients in 2005; ^b^ Survey among the general population in 2007.
