# Supplementary material for: Disentangling the dynamics of social assistance: A linked survey—Register data cohort study of long-term social assistance recipients in Norway
Source: PLoS One. 2020 Mar 27;15(3):e0230891. doi: 10.1371/journal.pone.0230891 (PMC7100955; doi:10.1371/journal.pone.0230891)
Supplement: S5 Table — Logistic regression analysis of social assistance, by (a) childhood disadvantages, (b) health status, (c) health behavior, (d) psychological resources, or (e) social ties, and covariates (age, age squared, gender, marital status, educational level, and country background). Average marginal effects (AME) shown. (DOCX) [file pone.0230891.s005.docx]

**S5 Table. Logistic regression analysis of social assistance, by (a) childhood disadvantages, (b) health status, (c) health behavior, (d) psychological resources, or (e) social ties, and covariates (age, age squared, gender, marital status, educational level, and country background). Average marginal effects (AME) shown.**

|  | Outcome: social assistance (SA) | | |
| --- | --- | --- | --- |
| Explanatory variable | (1)  SA in 2013 | (2)  1.5G SA Ever  (2005—2013) | (3)  SA Always  (2009—2013) |
| **(A) Childhood disadvantages** |  |  |  |
| Economic hardships | 0.052 (0.048) | 0.011 (0.048) | 0.032 (0.033) |
| Parental drug/alcohol prob. | 0.014 (0.054) | 0.009 (0.054) | -0.033 (0.038) |
| Sexual abuse | -0.028 (0.076) | 0.116 (0.072) | -0.035 (0.057) |
| Bullying (long-term) | 0.072 (0.051) | -0.009 (0.053) | 0.020 (0.036) |
| Attention problems school | 0.050 (0.054) | 0.088* (0.052) | -0.023 (0.036) |
| Moving | 0.040 (0.050) | -0.044 (0.050) | -0.012 (0.035) |
| **(B) Health status** |  |  |  |
| Psych. distress (HSCL-10) | 0.032 (0.033) | 0.106*** (0.031) | 0.034 (0.023) |
| Psych. wellbeing | -0.031 (0.021) | -0.027 (0.020) | -0.010 (0.014) |
| Experiences pain often | 0.018 (0.049) | 0.015 (0.048) | -0.014 (0.033) |
| Excellent/very good SRH | 0.005 (0.057) | -0.014 (0.058) | -0.013 (0.040) |
| Limiting illness (activities) | -0.064 (0.049) | 0.059 (0.047) | 0.014 (0.033) |
| Physical health (accomp.) | 0.010 (0.049) | -0.022 (0.049) | 0.008 (0.034) |
| **(C) Health behaviors** |  |  |  |
| Drinks often | 0.017 (0.063) | 0.098* (0.058) | 0.020 (0.041) |
| Alcohol problem | 0.107* (0.065) | 0.106* (0.061) | 0.016 (0.043) |
| Drug problem | 0.244*** (0.047) | 0.257*** (0.048) | 0.138*** (0.035) |
| Regular exercise (outdoors) | 0.044 (0.047) | 0.068 (0.047) | 0.029 (0.033) |
| Regular exercise (indoors) | 0.031 (0.060) | -0.001 (0.062) | 0.015 (0.041) |
| **(D) Psychological resources** |  |  |  |
| Mastery | 0.022 (0.032) | 0.069** (0.030) | 0.017 (0.021) |
| Self-worth | 0.047 (0.035) | 0.061* (0.035) | 0.012 (0.025) |
| Life satisfaction | -0.032 (0.021) | -0.046** (0.021) | -0.013 (0.015) |
| Work motivation | -0.045* (0.023) | -0.047** (0.023) | -0.029* (0.015) |
| **(E) Social ties** |  |  |  |
| Social capital | -0.025** (0.011) | -0.038*** (0.010) | -0.011 (0.008) |
| Seldom visits/visited | 0.129** (0.053) | -0.079 (0.056) | 0.033 (0.037) |
| Often lonely | 0.108** (0.047) | 0.095** (0.048) | 0.069** (0.033) |
| No close friends around | 0.027 (0.054) | 0.009 (0.053) | 0.006 (0.037) |
| Seldom meets friends | -0.064 (0.062) | -0.066 (0.060) | -0.013 (0.043) |
| Seldom meets siblings | -0.039 (0.052) | -0.020 (0.051) | -0.021 (0.036) |
| Seldom meets parents | -0.025 (0.057) | -0.063 (0.056) | -0.066 (0.043) |

Significance level: *** = 0.01 ** = 0.05 * = 0.1; Standard errors in parentheses; Only the coefficient for the explanatory variable(s) shown; The explanatory variables are included separately; All models controls for age (and age^2^), female, married/cohab., VGS/higher educ., and born abroad.
